# Insurance Reimbursement for Special Foods and Phenylalanine Levels in Patients With PKU in China

**DOI:** 10.1001/jamanetworkopen.2024.12886

**Published:** 2024-06-05

**Authors:** Bo Wang, Yanzhi Xia, Mingyue Cheng, Huili Luo, Luxi Xue, Anyue Gong, Xu Liu, Gaoqi Liao, Jieping Song, Kang Ning

**Affiliations:** 1 College of Life Science and Technology, Huazhong University of Science and Technology, Wuhan, Hubei, PR China; 2 Medical Genetics Center, Maternal and Child Health Hospital of Hubei Province, Wuhan, Hubei, PR China; 3 Department of Medical Laboratory Technology, Shiyan Maternal and Child Health Hospital, Affiliated Hospital of Hubei University of Medicine, Shiyan, Hubei, PR China; 4 Child Health Department, Maternal and Child Health Hospital of Jingzhou, Jingzhou, Hubei, PR China; 5 Newborn Disease Screening Laboratory, Xiangyang Maternal and Child Health Hospital, Xiangyang, Hubei, PR China

## Abstract

**Importance:**

Recent changes in China’s social medical insurance reimbursement policy have impacted the financial burden of patients with phenylketonuria (PKU) for special foods. However, whether this policy change is associated with their blood phenylalanine (PHE) concentration is unclear.

**Objective:**

To investigate the association between the reimbursement policy and blood PHE concentration in patients with PKU.

**Design, Setting, and Participants:**

This cohort study measured the blood PHE concentrations of 167 patients with PKU across 4 newborn screening centers in China from January 2018 to December 2021. The reimbursement policy for special foods for patients with PKU at 2 centers was canceled in 2019 and restored from 2020 onwards. In contrast, the other 2 centers consistently implemented the policy. Data were analyzed from September 10 to December 6, 2023.

**Exposures:**

The implementation and cancelation of the reimbursement policy for special foods of patients with PKU.

**Main Outcomes and Measures:**

The blood PHE concentration was regularly measured from 2018 to 2021. A 1-sided *Z* test was used to compare the mean of the blood PHE concentration between different years.

**Results:**

Among 167 patients with PKU (mean [SD] age, 84.4 [48.3] months; 87 males [52.1%]), a total of 4285 measurements of their blood PHE concentration were collected from 2018 to 2021. For patients at the center that canceled the reimbursement policy in 2019, the mean (SD) of the blood PHE concentrations in 2019 was 5.95 (5.73) mg/dL, significantly higher than 4.84 (4.11) mg/dL in 2018 (*P* < .001), 5.06 (5.21) mg/dL in 2020 (*P* = .006), and 4.77 (4.04) mg/dL in 2021 (*P* < .001). Similarly, for patients at the other center that canceled the policy in 2019, the mean (SD) of the blood PHE concentrations in 2019 was 5.95 (3.43) mg/dL, significantly higher than 5.34 (3.45) mg/dL in 2018 (*P* = .03), 5.13 (3.15) mg/dL in 2020 (*P* = .003), and 5.39 (3.46) mg/dL in 2021 (*P* = .03). On the contrary, no significant difference was observed between any of the years for patients at the 2 centers that consistently implemented the policy.

**Conclusions and Relevance:**

In this cohort study of patients with PKU from multiple centers, the implementation of the reimbursement policy for special foods was associated with controlling the blood PHE concentration. Special foods expenditure for patients with PKU should be included in the scope of long-term social medical insurance reimbursement.

## Key Points

**Question:**

Is social medical insurance spending on special foods for patients with phenylketonuria associated with their blood phenylalanine concentration?

**Findings:**

In this cohort study, the blood phenylalanine concentrations of 167 patients with phenylketonuria was assessed at multiple newborn screening centers in China from 2018 to 2021. Patients showed significantly higher blood phenylalanine concentrations during the year when the social medical insurance reimbursement policy for special foods was canceled, and concentrations decreased upon policy reinstatement.

**Meaning:**

These findings suggest that social medical insurance spending on special foods is crucial for patients with phenylketonuria to control their blood phenylalanine concentrations.

## Teaser

This cohort study examines the association between the reimbursement policy and blood phenylalanine concentration in patients with phenylketonuria in China.

## Introduction

Phenylketonuria (PKU) is a hereditary metabolic disease characterized by a deficiency of the enzyme phenylalanine hydroxylase, leading to reduced tyrosine levels and elevated phenylalanine (PHE) levels.^1^ The severity of the PKU varies depending on the concentration of PHE in tissues and blood, spanning from mild to severe manifestations.^1,2^ Poorly managed PKU during childhood can result in a diverse array of symptoms, including mental disorders, intellectual disability, behavioral issues, and seizures.^3^ Since 1981, the Chinese government has instituted a newborn screening program primarily targeting congenital hypothyroidism (CH) and PKU.^4^ PKU has exhibited an estimated overall incidence of approximately 1 of 11 144 newborns from 1985 to 2001.^4,5^

Treatment for PKU should commence within the first 10 days after birth, relying on prompt identification through newborn screening initiatives, which is crucial in preventing permanent harm, such as neurological issues and cognitive impairment.^6^ Moreover, continuing this management throughout an individual’s life is also essential for managing symptoms associated with brain function, such as difficulties with attention, working memory, and delayed reaction times.^7^ Despite patients with PKU being unable to metabolize PHE, they still require a small amount of food to meet their protein synthesis needs. Consequently, patients with PKU must adhere to a low-PHE diet and regularly monitor their blood PHE concentration.^1,7,8^ The special foods for patients with PKU include PHE-free protein substitutes and low-protein foods. Individuals with PKU typically have a blood PHE concentration of less than 2 mg/dL (to convert to μmol/L, multiply by 60.544), while patients with PKU who consume special foods should aim for a blood PHE concentration of less than 6 mg/dL.^8^

The financial expense of managing the diet of individuals with PKU is substantial, as families of patients with PKU typically purchase special foods regularly. They spend approximately ¥60 000 to ¥150 000 on these special foods annually, with an average expenditure of about ¥80 000 (to convert to USD, multiply by 0.14). However, social medical insurance reimbursement policies and other forms of social support can largely alleviate the financial burden on patients with PKU. In China, social medical insurance reimbursement can cover up to 70% of the costs of purchasing special foods for patients with PKU. Thus, the social medical insurance reimbursement policy needs to remain stable to alleviate the financial burden on PUK patients.

In 2019, the Hubei Newborn Screening Center (HNSC) and the Xiangyang Newborn Screening Center (XNSC) in China discontinued the social medical insurance reimbursement policy for special foods. However, in other years (eg, 2018, 2020, and 2021), social medical insurance was able to reimburse 70% of the expenses for special foods. On the contrary, the Shiyan Newborn Screening Center (SNSC) and Jingzhou Newborn Screening Center (JNSC) in China consistently implemented the social medical insurance reimbursement policy from 2018 to 2021. These changes provided a unique opportunity for us to investigate the associations between the social medical insurance reimbursement policy and the blood PHE concentrations of patients with PKU.

## Methods

The Ethics Committee of Maternal and Child Health Hospital of Hubei Province approved the research. The guardians of patients provided written and verbal informed consent. This cohort study followed the Strengthening the Reporting of Observational Studies in Epidemiology (STROBE) reporting guideline.

### Study Design

This cohort study assessed the fluctuation in blood PHE concentrations of patients with PKU from multiple newborn screening centers over 4 years (January 2018 to December 2021). The study involved the 4 largest newborn screening centers affiliated with tertiary care maternal and child health hospitals in Hubei Province, China, including HNSC, XNSC, SNSC, and JNSC. Among these 4 centers, the HNSC and XNSC continued supplying special foods throughout the 4 year period, and the prices remained unchanged despite the cancellation of reimbursement in 2019 and its restoration in 2020. The other 2 centers continued supplying special foods and implementing the policy throughout the study period. The special foods included PHE-free protein substitutes and low-protein foods.

The selection of these centers was guided by specific criteria: (1) policy variability (the cancellation and restoration of the social medical insurance reimbursement policy in 2 centers, while the other 2 centers consistently implemented policy throughout the study period, serving as a basis for comparison); (2) early establishment (HNSC was established in 2006, with the other 3 centers established before 2012); (3) large screened sample size (by the end of 2021, HNSC had screened over 2.5 million, Xiangyang 600 000, with the other 2 centers also screening more than 300 000 newborns); (4) adherence to operational standards (all centers were affiliated with tertiary care hospitals featuring comprehensive laboratory quality control); and (5) data integrity (all the 4 centers have included the presence of dedicated personnel for management, statistical data compilation, and the implementation of data management systems). The inclusion criteria of patients for this cohort study are as follows: (1) patients with PKU diagnosed through screening and confirmation at the 4 newborn screening centers; (2) blood monitoring conducted at the newborn screening centers over 4 years (2018 to 2021); (3) children aged between 0 and 14 years during the monitoring period; (4) measurement of PHE levels is only considered valid in this study if there have been no other internal or surgical illnesses in the 4 weeks preceding the measurements; and (5) willingness to participate in this study.

### Data Collection

The blood PHE concentration of patients with PKU was measured regularly. Data from patients with PKU spanning the years 2018 to 2021 were collected for the analysis. The instrument used for measurement is the Genetic Screening Processor for Neonatal Screening (Perkin Elmer, 2021-0010). The equipment, reagents, and personnel involved in this study are all appropriately qualified.^9^

### Statistical Analysis

The χ^2^ test, performed by the stats package in R version 4.1.2 (R Project for Statistical Computing), was used to compare the age or sex distribution across different centers. A 1-sided *Z* test, performed by the BSDA package in R version 1.2.2 (R Project for Statistical Computing), was used to compare the mean of the blood PHE concentration between different years. To assess the impact of policy adjustments on blood PHE concentration, we designated the year 2019, coinciding with the policy change, as the reference year. Subsequently, a mixed linear regression model was used to analyze samples collected from HNSC and XNSC centers, using the lme4 package in R version 1.1.28 (R Project for Statistical Computing). Specifically, the model considered random variability introduced by patients while estimating the fixed effects of year and center. Statistical significance was set at *P* < .05. Data were analyzed from September 10 to December 6, 2023.

## Results

### Baseline Characteristics of Study Participants

A total of 167 patients with PKU (mean [SD] age, 84.4 [48.3] months; 87 males [52.1%]) were enrolled in this study who had their blood PHE concentration monitored by 4 newborn screening centers, including HNSC (81 [48.5%]), XNSC (41 [24.6%]), SNSC (25 [15.0%]), and JNSC (20 [12.0%]). Table 1 presents participant characteristics at baseline. Regarding sex distribution, the overall male-to-female ratio was 108.8% (87 males to 80 females). Specifically, HNSC had a ratio of 107.7% (42 males to 39 females), XNSC at 115.8% (22 males to 19 females), SNSC at 108.3% (13 males to 12 females), and JNSC at 100.0% (10 males to 10 females). The sex distributions across the centers exhibited no significant difference (*P* > .99). Regarding the age distributions, the HNSC had 81 patients, with 5 (6.2%) in the 0 to 12 months old age group, 11 (13.6%) in the 13 to 36 months old group, 17 (21.0%) in the 37 to 72 months old group, and 48 (59.3%) in the 72 months or older group. The XNSC had 41 patients, with 3 (7.3%), 6 (14.6%), 10 (24.4%), and 22 (53.7%) in the respective age groups. The SNSC had 25 patients, distributed as 1 (4.0%), 4 (16.0%), 6 (24.0%), and 14 (56.0%) in the corresponding age categories. Lastly, the JNSC had 20 patients, with 2 (10.0%), 3 (15.0%), 5 (25.0%), and 10 (50.0%) in the respective age groups. The age distributions showed no significant difference (*P* > .99).

**Table 1.** Baseline Characteristics of Study Participants

Charateristic	Newborn screening center, No. (%)			
	Hubei (n = 81)	Xiangyang (n = 41)	Shiyan (n = 25)	Jingzhou (n = 20)
Age, mos				
0-12	5 (6.2)	3 (7.3)	1 (4.0)	2 (10.0)
13-36	11 (13.6)	6 (14.6)	4 (16.0)	3 (15.0)
37-72	17 (21.0)	10 (24.4)	6 (24.0)	5 (25.0)
>72	48 (59.3)	22 (53.7)	14 (56.0)	10 (50.0)
Sex				
Female	39 (48.1)	19 (46.3)	12 (48.0)	10 (50.0)
Male	42 (51.9)	22 (53.7)	13 (52.0)	10 (50.0)

### Insurance Reimbursement for Special Foods and Fluctuation of the Blood PHE Levels

A total of 4285 measurements of the blood PHE concentration were collected from 167 patients with PKU across 4 newborn screening centers from 2018 to 2021, including HNSC (2077 [48.5%]), XNSC (1015 [23.7%]), SNSC (688 [16.1%]), and JNSC (505 [11.8%]). Table 2 shows the number of measurements and the mean (SD) of the blood PHE concentrations in each year at 4 centers.

**Table 2.** The Blood PHE Concentration of Patients With PKU Monitored at 4 Centers From 2018 to 2021

Newborn screening center	No. of measurements, No. (%)	Blood PHE concentration, mean (SD), mg/dL
**Hubei (n = 2077)**		
2018	508 (24.5)	4.84 (4.11)
2019	514 (24.7)	5.95 (5.73)
2020	452 (21.8)	5.06 (5.21)
2021	603 (29.0)	4.77 (4.04)
**Xiangyang (n = 1015)**		
2018	224 (22.1)	5.34 (3.45)
2019	220 (21.7)	5.95 (3.43)
2020	275 (27.1)	5.13 (3.15)
2021	296 (29.2)	5.39 (3.46)
**Shiyan (n = 688)**		
2018	163 (23.7)	5.57 (4.14)
2019	197 (28.6)	5.50 (4.86)
2020	157 (22.8)	5.27 (3.79)
2021	171 (24.9)	5.46 (4.05)
**Jingzhou (n = 505)**		
2018	74 (14.7)	5.22 (4.48)
2019	129 (25.5)	5.48 (4.64)
2020	168 (33.3)	5.53 (4.47)
2021	134 (26.5)	5.40 (3.93)

Abbreviations: PHE, phenylalanine; PKU, phenylketonuria.

SI converstion factor: To convert phenylalanine to μmol/L, multiply by 60.544.

The Figure shows the variation in the blood PHE concentration of patients with PKU over 4 years. A comparison of PHE concentrations among years was conducted for each center. For patients at HNSC, the mean (SD) of the blood PHE concentrations in 2019 was 5.95 (5.73) mg/dL, significantly higher than 4.84 (4.11) mg/dL in 2018 (*P* < .001), 5.06 (5.21) mg/dL in 2020 (*P* = .006), and 4.77 (4.04) mg/dL in 2021 (*P* < .001). No significant difference was observed between other years except for 2019 (*P* > .15). Similarly, for patients at XNSC, the mean (SD) of the blood PHE concentrations in 2019 was 5.95 (3.43) mg/dL, significantly higher than 5.34 (3.45) mg/dL in 2018 (*P* = .03), 5.13 (3.15) mg/dL in 2020 (*P* = .003), and 5.39 (3.46) mg/dL in 2021 (*P* = .03). No significant difference was observed between other years except for 2019 (*P* > .15). Furthermore, considering the random variability introduced by patients, a mixed linear regression model was applied on samples from HNSC and XNSC sites. Using 2019 as the reference year, results showed that the mean differences between 2019 and the other years were significant (*P* < .001). In contrast, for patients at SNSC, no significant difference was observed (*P* > .20) in the mean (SD) of the blood PHE concentrations between any of the years: 5.57 (4.14) mg/dL in 2018, 5.50 (4.86) mg/dL in 2019, 5.27 (3.79) mg/dL in 2020, and 5.46 (4.05) mg/dL in 2021. For patients in JNSC, no significant difference was observed (*P* > .30) in the mean (SD) of the blood PHE concentrations between any of the years: 5.22 (4.48) mg/dL in 2018, 5.48 (4.64) mg/dL in 2019, 5.53 (4.47) mg/dL in 2020, and 5.4 (3.93) mg/dL in 2021.

**Figure.**
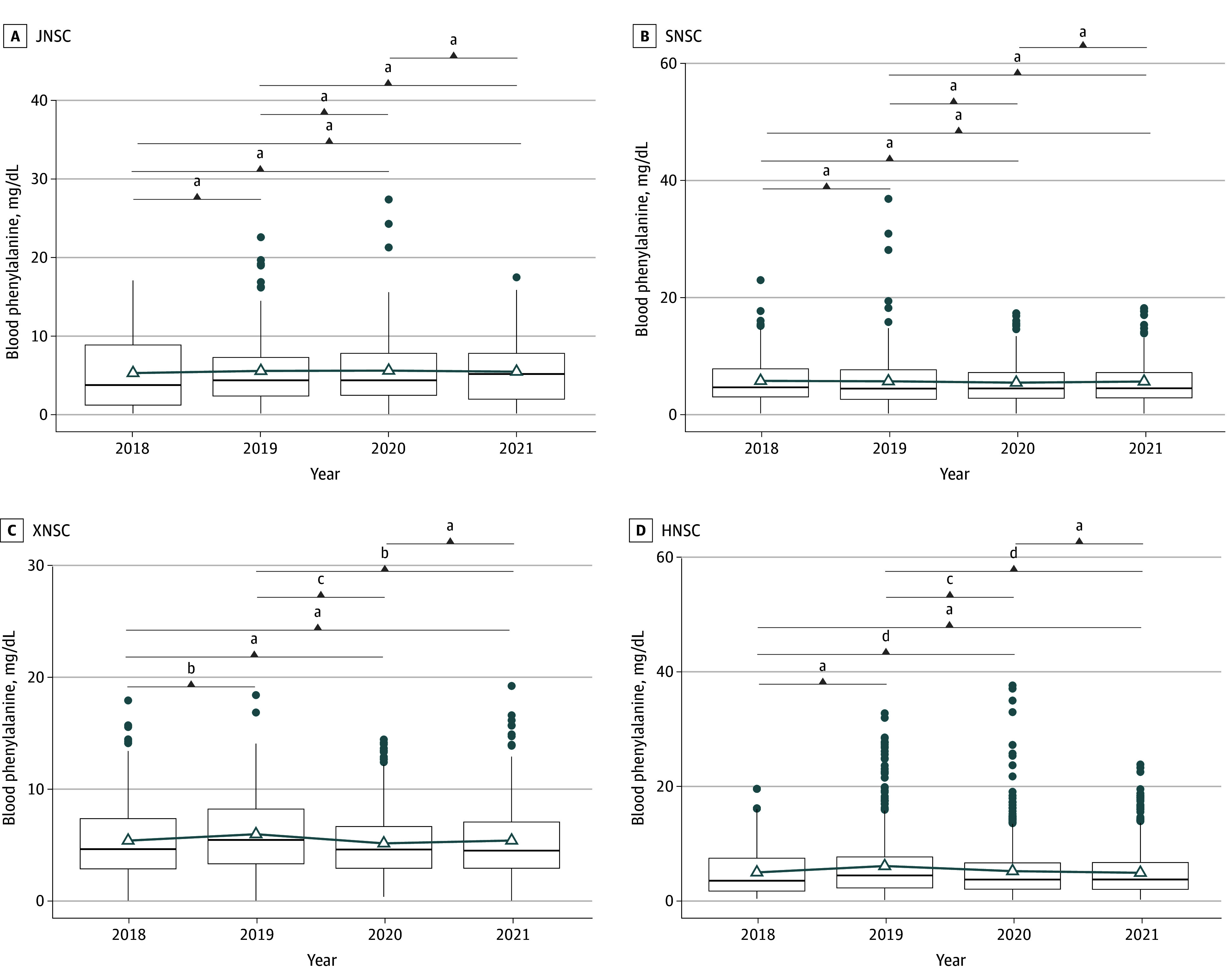
Temporal Dynamics of the Blood Phenylalanine Concentration Monitored at 4 Centers From 2018 to 2021 Boxplots indicate the differences in blood phenylalanine concentration (mg/dl) across 4 years in 4 hospitals. The mean values of each year are shown in triangle dots and connected with lines to show the variation trend. For all the boxplots, boxes represent the IQR between the first and third quartiles, and the line inside represents the median. Whiskers denote the lowest and highest values within the 1.5 × IQR from the first and third quartiles, respectively. Statistical significance was tested using the 1-sided *Z* test. HNSC indicates Hubei Newborn Screening Center; JNSC, Jingzhou Newborn Screening Center; SNSC, Shiyan Newborn Screening Center; XNSC, Xiangyan Newborn Screening Center. To convert phenylalanine to μmol/L, multiply by 60.544. ^a^Not significant. ^b^*P* < .05. ^c^*P* < .01. ^d^*P* < .001.

These results suggest that blood PHE concentration of patients with PKU at HNSC and SNSC was associated with an increase in the year when insurance reimbursement for special foods was canceled and subsequently decreased in the following year when the reimbursement was restored. However, for patients with PKU at the other 2 centers, their blood PHE concentration remained relatively stable across the 4 years. These findings imply that the implementation or absence of insurance reimbursement for special foods may be associated with fluctuations in the blood phenylalanine levels of patients with PKU.

## Discussion

Certain countries have established subsidies or health insurance systems to cover some or all of the special foods used by patients with PKU, including PHE-free protein substitutes and low-protein foods.^8,10,11^ As previously reported,^8^ European countries, including Turkey, Poland, the UK, Belgium, Denmark, Italy, Norway, and Spain, fully reimbursed protein substitutes. In Germany and the Netherlands, the costs are covered by health insurance. In contrast, special low-protein foods varied from being fully reimbursed to self-payment across these countries. For instance, only the governments of the UK and Italy fully reimbursed low-protein foods via a national prescription system. Patients in Belgium, Denmark, Norway, and Turkey received monthly or annual financial allowances for special low-protein foods. Unlike these countries, in China, the cost of PHE-free protein substitutes and low-protein foods can be covered up to 70% by the social medical insurance reimbursement policy. However, there have been fluctuations in this reimbursement policy in recent years. For instance, in 2019, the policy was canceled in Wuhan and Xiangyang in China and restored from 2020 onwards.

This cohort study encompassed 4 centers in China. The HNSC was responsible for monitoring blood PHE concentration and selling special foods to patients with PKU aged 0 to 14 years in Wuhan city and surrounding areas. The other 3 newborn screening centers also carried out the same tasks in their respective cities (Shiyan, Jingzhou, and Xiangyang). At these 4 centers, the selling prices of phenylalanine-free protein substitutes closely aligned with the manufacturer’s selling price, and the prices and types of special foods at these centers remained unchanged over the 4 years (2018 to 2021). Analysis of the study data showed that in 2019, when the reimbursement policy for special foods was canceled, an increase in blood PHE concentration among patients with PKU from HNSC and XNSC was observed, and a decrease in blood PHE concentration was observed after the policy was restored in the followed years. Conversely, in SNSC and JNSC, the annual averages of blood PHE concentration values remained relatively stable from 2018 to 2021, during which the reimbursement policy was consistently implemented.

These findings suggested that canceling the social medical insurance for special foods was associated with increased blood PHE concentration of patients with PKU. Such an increased clinical burden might exacerbate the risk of various comorbidities,^11^ thus imposing additional social and economic burdens on patients with PKU. Contemporary literature,^12,13,14,15^ coupled with our analytical results, underscores the critical importance of maintaining stable social medical insurance reimbursement policies. Historically, before 1977, dietary PHE restriction was deemed necessary only during early childhood. However, current understanding emphasizes the need for prolonged dietary management throughout life,^16^ particularly for pregnant females with PKU to prevent adverse effects on fetal nervous system development. Moreover, reintroduction of the low-PHE diet among patients with PKU who have discontinued it often proves challenging.^17^ This study highlights the importance of implementing long-term support strategies, such as adaptive insurance policies tailored to the lifelong needs of patients with PKU, and ensuring continuous access to necessary dietary treatments and regular monitoring.

### Limitations

This study has limitations. First, the inclusion of more patients in China may reinforce the conclusions. However, we cannot arbitrarily alter medical insurance reimbursement health care policies to set more comparison groups. Second, the presence of the complex influencing factors in daily life that may affect the PHE concentration of patients over the 4 years cannot be entirely removed in the analysis. Despite these limitations, the noticeable increase in PHE concentrations following the cancellation of insurance reimbursement underscores the need for long-term insurance policies for special foods.

## Conclusions

In this cohort study of patients with PKU, the implementation of the reimbursement policy for special food expenditure of patients with PKU was associated with controlling their blood PHE concentrations. Considering contemporary Chinese economics and income levels, the special food expenditure of patients with PKU should be included in the scope of social medical insurance reimbursement.

## Notes

Supplement.
Data Sharing Statement
