# Supplementary material for: Insurance Reimbursement for Special Foods and Phenylalanine Levels in Patients With PKU in China
Source: JAMA Netw Open. 2024 Jun 5;7(6):e2412886. doi: 10.1001/jamanetworkopen.2024.12886 (PMC11154153; doi:10.1001/jamanetworkopen.2024.12886)
Supplement: Supplement. — Data Sharing Statement [file jamanetwopen-e2412886-s001.pdf]

Data Sharing Statement

Wang. Insurance Reimbursement for Special Foods and Phenylalanine Levels in Patients With PKU in China. *JAMA Netw Open*. Published June 04, 2024.
doi:10.1001/jamanetworkopen.2024.12886

Data
Data available: No
